# Accelerated Low-Dose Total Skin Electron Beam Therapy Using the Modified Stanford Technique: An In Vivo Dosimetry Confirmation Study

**DOI:** 10.7759/cureus.75422

**Published:** 2024-12-09

**Authors:** Omar El Fadel, Leland Damron, Christian Fernandez, Louis Cappelli, Karen Mooney, Wenyin Shi

**Affiliations:** 1 Radiation Oncology, Thomas Jefferson University Hospital, Philadelphia, USA; 2 Physics, Thomas Jefferson University Hospital, Philadelphia, USA

**Keywords:** cutaneous t cell lymphomas (ctcl), modified stanford technique, mycosis fungoides, optically stimulated radiation dosimeters, total skin electron beam therapy

## Abstract

Purpose

Low-dose total skin electron beam therapy (LD-TSEBT) has recently gained popularity in treating mycosis fungoides (MF) due to its reduced toxicity and favorable response rates. Combining accelerated LD-TSEBT with the modified Stanford technique (mST), a condensed cycling approach, offers a promising and convenient option. However, in vivo dosimetry data confirming the effectiveness of this approach is limited. We retrospectively analyzed in vivo data from patients who received accelerated LD-TSEBT using the mST for MF.

Methods

Patients treated with accelerated LD-TSEBT using the mST for MF were identified. Optically stimulated radiation dosimeters (OSLDs) were used to measure doses at 10 anatomical sites: vertex, larynx, right shoulder, right forearm, right hip, umbilicus, left medial thigh, right knee, left dorsal foot, and left dorsal hand. Measurements were aggregated and compared to the prescribed dose, using the European Organisation For Research And Treatment Of Cancer (EORTC) homogeneity tolerance criteria, which account for the American Association of Physicians in Medicine (AAPM) TG023 setup variability: ±20% of the prescribed dose. Patient characteristics, demographics, and disease details were also collected. Descriptive statistics were performed to evaluate clinical and dosimetric characteristics.

Results

Thirty-six patients were identified, and 360 OSLD measurements were recorded. The median of all OSLD measurements relative to the prescribed dose at all sites was 97.4%. The highest median delivered dose was recorded at the umbilicus (106%) and the lowest at the left dorsal hand (79%). After accounting for deviation at the left dorsal hand, 85.8% of all OSLD measurements met the homogeneity criteria at the other anatomic sites. Other patient metrics, such as height and BMI, did not impact the median delivered OSLD dose or number of anatomical sites per patient meeting the EORTC tolerance criteria.

Conclusion

Accelerated LD-TSEBT using the mST delivers accurate doses, with most subjects meeting the EORTC tolerance criteria. This study supports the use of OSLDs for in vivo dosimetry in patients undergoing this regimen, ensuring adequate dosing despite the truncated cycling approach.

## Introduction

Total skin electron beam therapy (TSEBT) is a proven therapeutic modality for the treatment of cutaneous T-cell lymphoma (CTCL), particularly mycosis fungoides (MF). Historically, high-dose regimens (~36 Gy) have been associated with significant toxicity, such as desquamation and alopecia, making them less feasible for frail patients [1,2]. More recent approaches, like low-dose TSEBT (LD-TSEBT), have demonstrated efficacy in reducing disease burden while minimizing toxicity [3-5]. Accelerated LD-TSEBT further streamlines treatment, offering similar benefits in a condensed schedule, typically over two weeks [5,6]. To ensure the safe and effective delivery of TSEBT, dosimetric accuracy and quality control are essential [7-9].

In vivo dosimetry is crucial for verifying dose delivery during TSEBT. Techniques such as thermoluminescent dosimeters (TLDs), optically stimulated luminescence dosimeters (OSLDs), and Gafchromic® (Ashland Global, Wilmington, DE, United States) film dosimetry have been used in various clinical studies [10-14]. However, challenges such as dose inhomogeneity, particularly in the peripheral areas, and variability due to patient factors (e.g., BMI and height) necessitate meticulous monitoring and optimization [8-12]. This study evaluates the efficacy and dosimetric accuracy of accelerated LD-TSEBT using the modified Stanford technique (mST) device, focusing on compliance with established quality criteria and the impact of patient characteristics on dose delivery.

## Materials and methods

Methods

This study was conducted at Thomas Jefferson University Hospital, a tertiary academic medical center in Philadelphia. We retrospectively reviewed all patients diagnosed with MF who received accelerated LD-TSEBT using the modified Stanford VI dual-field technique between 2015 and 2021. Patients were identified, and information was extracted from electronic medical records, including demographics, disease characteristics, BMI, prior treatments, radiotherapy treatment specifics, and treatment outcomes. The hospital's institutional review board (IRB) evaluated the study and granted a waiver for informed consent under Title 45 Code of Federal Regulations Part 46.116(D) (#18D.480). 

Radiation therapy

**Figure 1:**
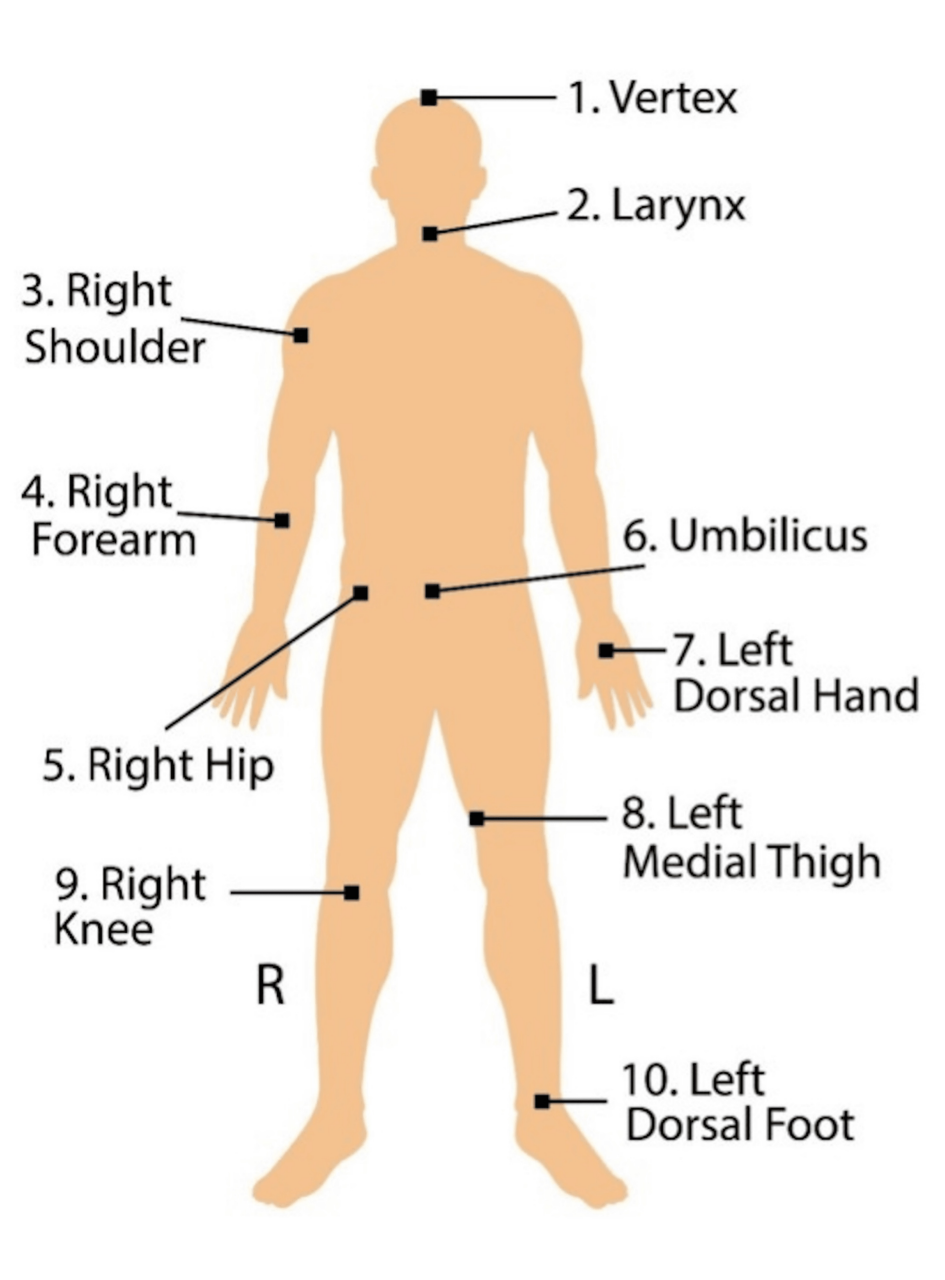
The 10 anatomical sites

The modified Stanford VI dual-field technique has been previously described [5]. All patients received a dose of 12 Gy in 2-Gy daily treatments, one complete cycle per day, and three fractions per week. Treatments were delivered using a high-dose rate (25 Gy/min) 6-MeV electron beam at an extended source‐to‐surface distance (SSD) of 390 cm with an acrylic spoiler. Six patient positions were utilized during each treatment: upper and lower anterior-posterior/posterior-anterior, left anterior oblique/right anterior oblique, and left posterior oblique/right posterior oblique. NanoDot™ (Landauer, Inc., Glenwood, IL, United States) optically stimulated luminescence dosimeters (OSLDs) were placed on the patient and remained in position throughout all 12 treatment fields for dose measurements [6]. OSLDs were used at 10 anatomical sites: vertex, larynx, right shoulder, right forearm, right hip, umbilicus, left dorsal hand, left medial thigh, right knee, and left dorsal foot (Figure 1). At each patient position, two large (40 cm x 40 cm) fields were used, with the linac gantry directed above and below the patient's body (Gantry 252°/288°) to ensure full superior-to-inferior coverage of the patient and reduce photon dose contamination. 

Statistical analysis

OSLD measurements were aggregated, analyzed, and compared to the prescribed dose. Average OSLD readings at all measurement sites were compared to the prescribed dose based on the American Association of Physicists in Medicine Task Group 023 (AAPM TG-23, ±10% setup/readout variability) and the European Organization for Research and Treatment for Cancer (EORTC, ±10% homogeneity) tolerances (±20% total).

Descriptive statistics were used to evaluate patient and individual OSLD measurements. ANOVA testing was performed to assess the impact of BMI categories on the median of the 10 OSLD measurements per patient and the number of anatomical sites within tolerance per patient. Linear regression was applied to evaluate the impact of height on the median of the 10 OSLDs measurement per patient and the number of anatomical sites within tolerance. Analysis was performed using Microsoft Excel (Microsoft Corp., Redmond, WA, United States) and GraphPad Prism (Dotmatics, Boston, MA, United States).

## Results

Patient characteristics

A total of 36 patients met the inclusion criteria and were included in this retrospective analysis. The median age was 66.5 years (range, 29-89 years). Ten patients (28%) were female, 47% identified as White patients, and 42% identified as Black patients. The median BMI for the study group was 28.5 kg/m² (range, 17.1-44.7 kg/m²), and the median height was 1.75 m (range, 1.50-2.03 m). At the time of TSEBT, 25 patients were diagnosed with stage I or II disease, and nine patients were diagnosed with stage III or IV disease (Table 1).

**Table 1:** Patient and disease characteristics

Variable	Total	%/range
Patients	36	100
Sex		
Male	26	72
Female	10	28
Age, median	66.5	29-89
Race/ethnicity		
White	17	47
Black	15	42
Other	4	11
Measurements		
Height (m), median	1.8	1.5-2.0
Weight (kg), median	90.3	48.1-114.8
BMI (kg/m^2^), median	28.5	17.1-44.7
Stage at TSEBT, n (%)		
I/II	25	73.5
III/IV	9	26.5

OSLD measurements

**Figure 2:**
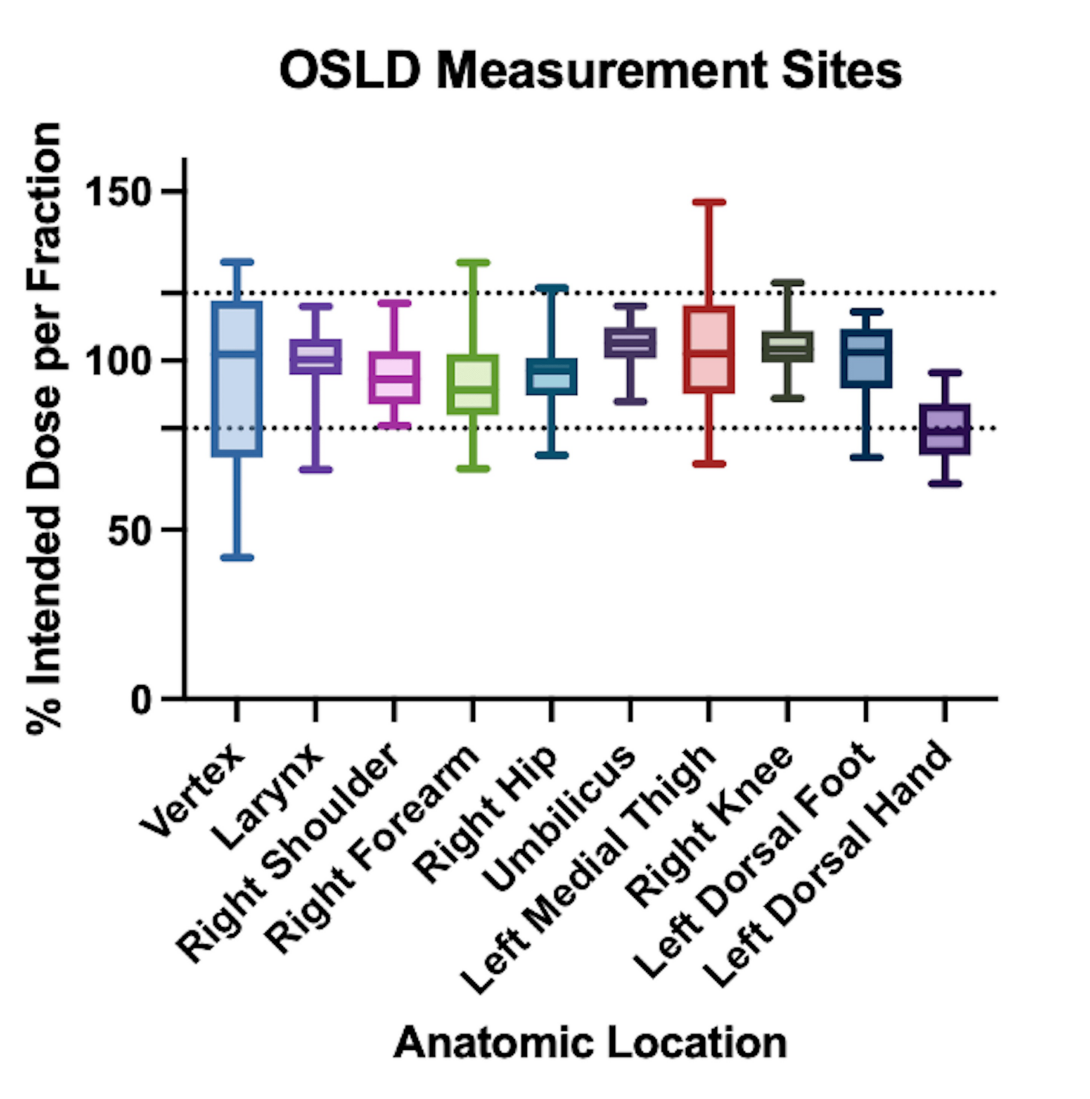
Measured dose and range as a percentage of intended prescribed dose across anatomical locations

A total of 349 OSLD measurements were recorded at 10 anatomical locations. Measurements for shielded anatomical areas were excluded from the analysis. The average OSLD measurement, relative to the prescribed dose, was 97.4% (range, 41.9%-146.8%), with the average per patient at 97.2% (range, 85.1%-115.3%) of the prescribed dose. A summary of the results by anatomical location is presented in Table 2 and Figure 2. Among the 10 anatomical locations across patients, the median OSLD measurement for the umbilicus was the highest, at 106% of the prescribed dose. The umbilicus also had the lowest variability, with a range of 88%-116%. Conversely, the left dorsal hand had the lowest median OSLD measurement at 79% (range, 63.7%-96.5%), representing the least accurate site. The vertex measurements exhibited the greatest degree of variability, with a range of 42%-129%. Of all registered OSLD measurements, 87.7% fell within the TG-023 and EORTC tolerances. When evaluating the number of OSLD measurements per patient, the average percentage within tolerance was 81.8% (range, 44.4%-100%). After excluding the left dorsal hand measurements due to known deviation from the target dose, 85.8% of OSLD measurements per patient (range, 50.0%-100.0%) were within tolerances.

**Table 2:** Summary of OSLD measurements expressed as percent of intended dose across different anatomical locations

Variable	N	Median	Average	STD	Range
Vertex	28	102%	95%	25%	42%-129%
Larynx	36	100%	99%	11%	68%-116%
Right shoulder	36	94%	96%	10%	81%-117%
Right forearm	36	92%	94%	17%	68%-129%
Right hip	36	97%	97%	11%	72%-122%
Umbilicus	36	106%	105%	7%	88%-116%
Left medial thigh	34	102%	104%	18%	70%-147%
Right knee	35	103%	105%	8%	89%-123%
Left dorsal foot	36	102%	100%	11%	71%-115%
Left dorsal hand	36	79%	80%	9%	64%-97%

BMI and height

**Figure 3:**
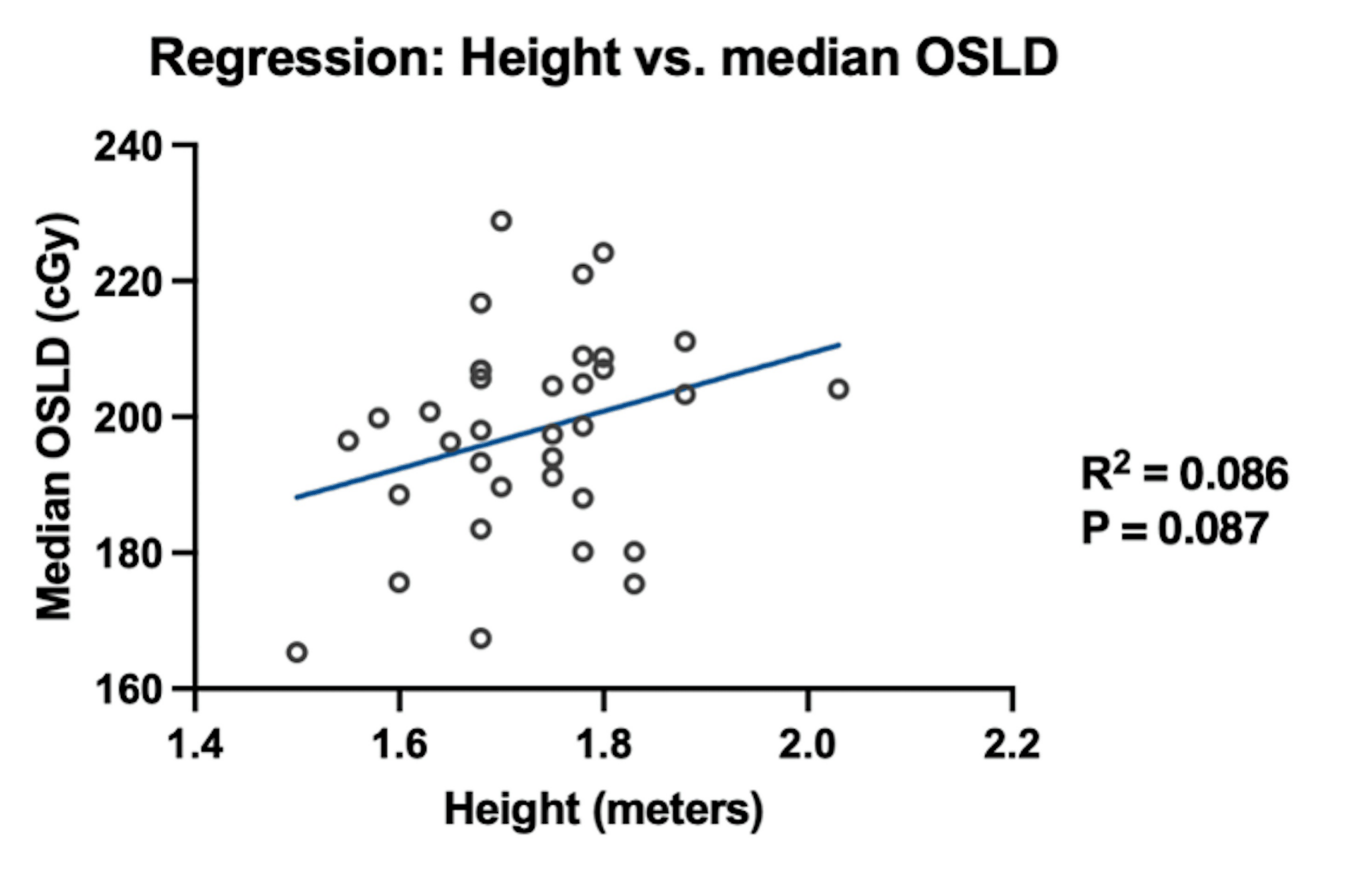
Regression between median OSLD measurement per patient (cGy) and height (meters)

**Figure 4:**
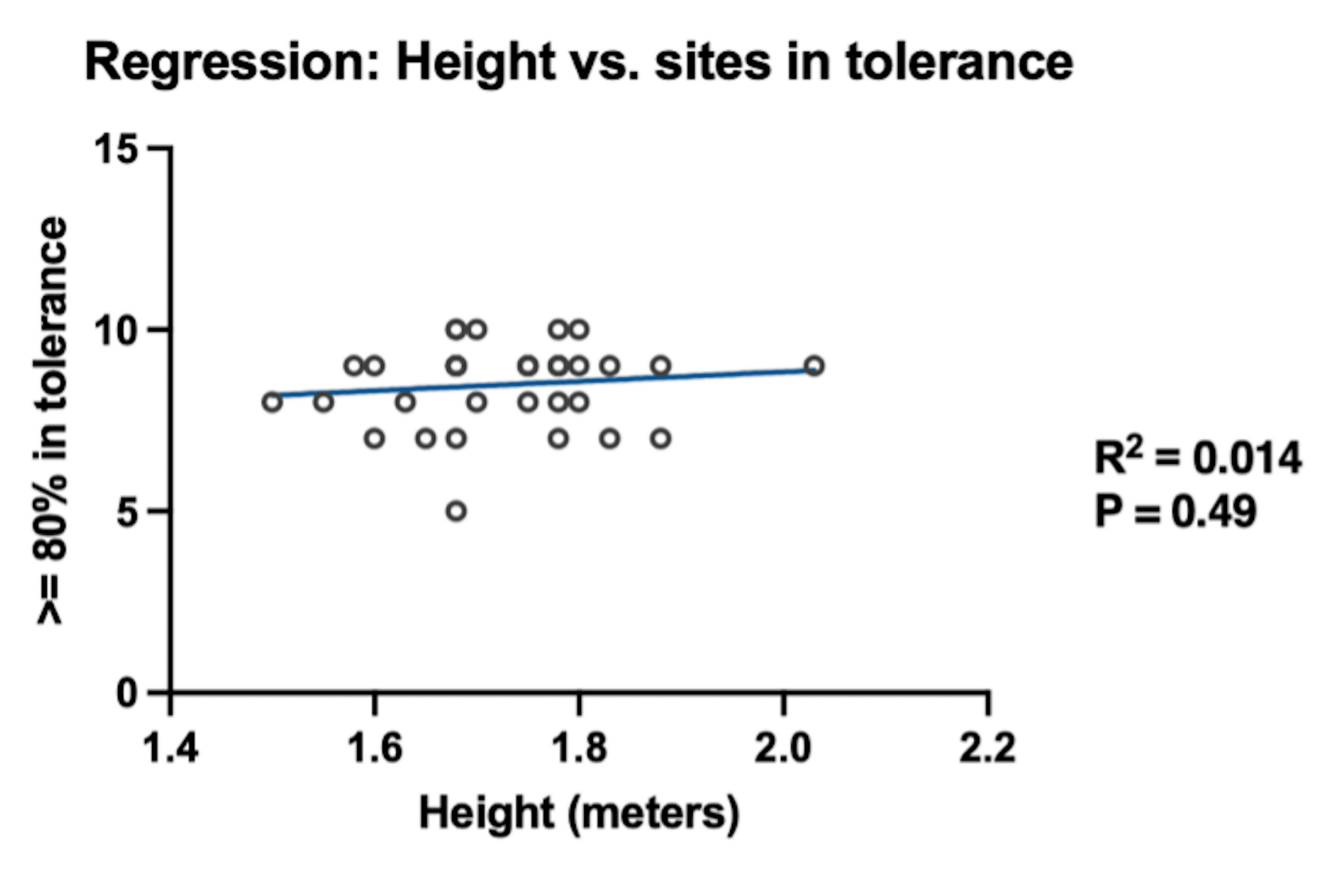
Regression between the number of anatomical sites within 80% in tolerance and height in meters

The patients were divided into three groups by BMI: (1) BMI < 25 kg/m² (n = 7), (2) BMI 25-30 kg/m² (n = 14), and (3) BMI > 30 kg/m² (n = 13). Two patients were excluded from this analysis due to undocumented height and weight. The mean percentage of the intended OSLD dose for each BMI group is presented in Table 3. The mean OSLD per patient did not differ significantly across the three BMI subgroups (p = 0.16; Table 3). Additionally, there was no significant difference in the number of anatomical sites per patient that met 80% of the intended dose across the BMI subgroups (p = 0.83; Table 3). Neither the median OSLD measurement per patient (p = 0.09; Figure 3) nor the number of anatomical sites per patient meeting tolerance (p = 0.49; Figure 4) was associated with height.

**Table 3:** Mean percent intended OSLD dose across distinct BMI groups (BMI < 25, BMI 25-30, and BMI > 30)

Characteristic	N	Average dose (%Rx)	ANOVA p-value	ANOVA f-value	Average number of sites in tolerance	ANOVA p-value	ANOVA f-value
BMI < 25 (kg/m^2^)	7	103%	0.16	1.97	87%	0.83	0.18
BMI 25-30 (kg/m^2^)	14	99.2%			85%		
BMI > 30 (kg/m^2^)	13	96.70%			84%		

## Discussion

Accelerated LD-TSEBT using the mST is designed to provide effective and convenient treatment with reduced toxicity. Due to the inherent variability in TSEBT treatments, the EORTC and the AAPM have provided guidelines to ensure quality interventions [6,7]. These guidelines, taken together, indicate that OSLD measurements should be within ±20% of the prescribed dose. A sample from each NanoDot lot was irradiated under 100 cGy reference dose conditions. Any lot that did not meet an accuracy of ≤5% was returned to the vendor to ensure acceptable uncertainty in the OSLD measurements.

Prior publications have demonstrated the utility of in vivo dosimetry and the potential for variability in dose delivery during TSEBT [14]. Anacak et al. reviewed in vivo dosimetric data for 67 TSEBT treatments using six positions and measured at 10 different body points with thermoluminescent dosimetry (TLD) [8]. The authors noted around a 15% dose inhomogeneity throughout the skin, with the greatest variability in the extremities. However, details regarding dosing and cycling were not provided. Antolak et al. reported the in vivo data for 72 cases of MF treated with the mST to a standard dose of 32 Gy over eight weeks, measured at 22 body points [10]. Mean doses at body points, as a percentage of prescribed doses, were mostly between 74% and 98%, with standard deviations ranging from 4% to 23%. Analysis of dose accuracy relative to BMI demonstrated a significant correlation at some, but not all, body points. Baba et al. performed TSEBT in vivo dose verification for six patients, with 154 measurements using Gafchromic® EBT3 film strips [9]. Dose variation of up to 16% was observed, most notably on the hands. Regimens of 2 Gy per cycle were used, but further details were not provided. Guidi et al. conducted a literature review of TSEBT in-vivo dosimetry using various techniques [13]. They highlighted the impact of anatomical location, BMI, and patient positioning on variability.

Our study's mean OSLD measurement across anatomical sites per patient was 97.4%. Of all reported anatomical OSLD measurements, 87.7% of the measurements per patient met the EORTC and TG023 quality metrics [6,7]. Furthermore, the site with the greatest deviation from the intended dose was the left dorsal hand, which is consistent with the inherent high setup variability at this anatomical location. This finding aligns with other studies, where peripheral areas, such as the hands, were less accurate compared to other body areas [9,12,14]. After accounting for the left dorsal hand deviation, 85.8% of OSLD measurements were within tolerances. This is consistent with, if not better, previously published in vivo dosimetry data in TSEBT [8-14]. Given the high compliance rates within established quality criteria, this study supports the reliability of accelerated LD-TSEBT with the mST in treating MF. Furthermore, our results highlight that certain anatomical sites are more susceptible to variability due to setup factors, underscoring the need for closer monitoring in future clinical settings.

Given the truncated cycling and fractionation regimen associated with accelerated LD-TSEBT, ensuring adequate dosing throughout the treatment course is crucial. Patient characteristics, such as height and BMI, could influence homogenous dosing, as these factors may affect patient positioning relative to the gantry [8,11,13-14]. We hypothesized that patients with higher heights and BMIs might receive lower treatment OSLD measurements compared to the prescribed dose, based on prior work demonstrating a correlation between dose distribution and patient's gender, height, and weight [11,13]. Yet, our results found no such correlation. These findings reaffirm the adequacy of accelerated LD-TSEBT in delivering appropriate radiation doses despite variability in patient habitus and highlight the importance of in vivo dosimetry for evaluating dose and setup accuracy.

Several factors limit this study. First, data were collected from a single medical center in the Northeastern United States, which may limit the generalizability of our findings to a broader population, particularly as our study includes a larger proportion of male and White patients. In addition, while 349 measurements provide valuable insight, the overall sample size remains relatively small, which may reduce the power to detect certain significant correlations. Future studies should aim for a more diverse and extensive sample, incorporating patients from multiple institutions.

## Conclusions

Accelerated LD-TSEBT with the mST delivers accurate and convenient treatment. The use of OSLDs for in vivo dosimetry ensures reliable monitoring, enabling homogeneous and adequate dosing regardless of patient habitus.
